# Quality Assessment in FDG-PET/CT Imaging of Head-and-Neck Cancer: One Home Run Is Better Than Two Doubles

**DOI:** 10.3389/fonc.2020.01458

**Published:** 2020-08-14

**Authors:** Tim Van den Wyngaert, Stijn De Schepper, Laurens Carp

**Affiliations:** ^1^Department of Nuclear Medicine, Antwerp University Hospital, Edegem, Belgium; ^2^Faculty of Medicine and Health Sciences, University of Antwerp, Antwerp, Belgium

**Keywords:** FDG-PET/CT, quality, harmonization, quantification, head and neck cancer

## Abstract

2-deoxy-2-[^18^F]fluoro-D-glucose (FDG) positron emission tomography (PET)/computed tomography (CT) is indicated in head-and-neck cancer for the initial workup when clinically indicated (e. g., large tumors, clinically positive neck, cervical adenopathy from an unknown primary, etc.), for the assessment of treatment response 12 weeks after completion of (chemo)radiotherapy, and during follow-up when there is suspicion of relapse. The successful implementation of FDG-PET/CT in routine clinical practice requires an in-depth understanding of the recent advances in physics and engineering that have significantly improved the imaging capabilities of PET/CT scanners (e.g., digital silicon photomultipliers, point-spread function modeling, and time-of-flight, and Bayesian penalized likelihood reconstruction). Moreover, a coordinated harmonization effort from professional societies (e.g., EANM) and international bodies (e.g., IAEA) has resulted in the creation of quality assurance frameworks (e.g., QUANUM, EARL, GMP) and guidelines that collectively cover the entire spectrum from tracer production, hardware calibration, patient preparation, and scan acquisition, to image interpretation (e.g., PERCIST, Hopkins criteria). The ultimate goal is to standardize the PET/CT technique and to guarantee accurate and reproducible imaging results for every patient. This review summarizes the recent technical breakthroughs in PET/CT scan design and describes the existing quality assessment frameworks with a focus on applications in head-and-neck cancer. Strict adherence to these harmonization efforts will enable leveraging the full potential of PET/CT and translate the proven benefits of this technique into tangible improvements in outcome for patients with head-and-neck cancer in routine clinical care.

## Introduction

2-deoxy-2-[^18^F]fluoro-D-glucose (FDG) positron emission tomography (PET)/computed tomography (CT) is a hybrid functional imaging technique that visualizes tumor glucose metabolism. In head-and-neck cancers, the use of FDG-PET/CT is supported by the National Comprehensive Cancer Network (NCCN) and other societies for the initial workup when clinically indicated (e.g., large tumors, clinically positive neck, cervical adenopathy from an unknown primary, etc.). Also, the technique is recommended for the assessment of treatment response 12 weeks after completion of (chemo)radiotherapy, and during follow-up when there is suspicion of relapse (1). Subsequent technological advances in both PET and CT devices over the last decade have resulted in significant improvements in the imaging capabilities of the latest generation of integrated PET/CT scanners, resulting in improved sensitivity, higher image resolution, and important reductions in patient radiation exposure.

In routine clinical practice PET/CT images are usually reported using a strictly visual interpretation. Yet, there is increasing interest in leveraging the intrinsically quantitative nature of PET data. The use of standardized uptake values (SUV) is however prone to errors introduced by various factors. Therefore, a thorough understanding of these potential pitfalls is increasingly important to avoid erroneous interpretation and conclusions. This is also apparent in the setting of multicenter imaging trials, where patients are scanned across many sites using various scanners (2–4). In order to overcome the limitations of scanner and reconstruction specific SUV values, a coordinated effort of harmonization has been conducted to standardize the FDG-PET/CT technique. In parallel, a broader initiative to develop quality management in nuclear medicine has also contributed to improving the standard of care.

The aim of this narrative review is to highlight the various quality measures that exist today, focusing on the use of FDG-PET/CT in head-and-neck cancer. When possible the impact of these procedures will be illustrated with real-world evidence. This text is not intended to be exhaustive or a detailed recipe for high-quality FDG-PET/CT imaging, but rather a gentle introduction to the underlying critical concepts and frameworks.

## Methods

A best evidence review was performed by searching the PubMed database for English language publications indexed up to August 2019 using the keywords “positron emission tomography,” “PET,” “quality,” “harmonization,” and “FDG.” The abstracts of all 94 results were screened to identify publications addressing the technical basis supporting the need for harmonization, existing frameworks to perform standardized FDG-PET/CT imaging, and clinical data illustrating the impact of the use of these guidelines on reporting outcomes. Full-text published sources were preferred over abstract-only publications, whenever possible. Selected publications were screened for secondary references.

## Appropriate Use Criteria for FDG-PET/CT Imaging in Head-and-neck Cancer

Over the last decade, clinical trials have contributed to better defining the place of FDG-PET/CT in head-and-neck cancer, in particular in identifying the impact on patient management and outcomes. In particular, the prospective, randomized, controlled PET-NECK trial demonstrated non-inferior overall survival outcomes when FDG-PET/CT surveillance was performed 12 weeks after the end of chemoradiotherapy. In the FDG-PET/CT arm of the study, neck dissection was only performed if incomplete or equivocal response was seen on FDG-PET/CT, in contrast with planned neck dissection in patients with stage N2 or N3 disease in the comparator arm (5). In addition, FDG-PET/CT was also shown to be the more cost-effective strategy and associated with fewer complications than neck dissection (6).

These findings were quickly incorporated in the imaging recommendations of various societies, including the previously mentioned NCCN alliance (1). For example, the United Kingdom national multidisciplinary guidelines on imaging in head-and-neck cancer now conclude that currently a negative, normal FDG-PET/CT 12 weeks post-treatment likely offers the best prognostic reassurance (7). Also, it endorses the use of the technique to evaluate patients with malignant cervical adenopathy from an unknown primary as up-front indication, with a detection rate of an occult primary in approximately one third of cases. According to the same guideline, FDG-PET/CT is also valuable in the assessment of suspected recurrence of head-and-neck cancer when there are extensive, confounding post-treatment changes on conventional imaging modalities. Of note, the 12 week interval between the end of radiotherapy and FDG-PET/CT imaging to allow the resolution of inflammatory changes is now firmly established (8).

Similarly, a quality initiative from the Belgian Health Care Knowledge Centre concluded in 2015 that FDG-PET/CT is not recommended for the evaluation of metastatic spread and/or the detection of second primary tumors in patients with stage I–II squamous-cell head-and-neck tumors, while it is recommended for patients with stage III–IV disease (9). Nevertheless, a 2019 follow-up study noted that the use of this imaging technique in stage I–II patients was still 23%, well above the appropriate use target. Conversely, the same study found that only 48% of stage III-IV patients were offered FDG-PET/CT imaging for their disease, constituting a dramatic underuse (10). These findings underline the challenges of implementing existing guidelines in routine practice, which hampers improvements in patient outcomes.

## Recent Technological Advances in PET/CT Imaging

This section together with Table 1 presents a more technical overview of the recent advances in PET/CT physics and engineering for the interested reader, but can be skipped without loss of continuity.

**Table 1:** Impact of recent technical breakthroughs on PET/CT image quality.

**Technical advance and image impact**
**Digital silicon photomultiplier (SiPM)** •** Increased sensitivity** results in better statistics and less noise in the image. Equivalent image quality can be achieved with less activity administered to the patient, without increasing the scan time. • The increased sensitivity allows the use of smaller voxels without significant increase in noise related to limited statistics. This results in an **increased spatial resolution** and **signal-to-noise ratio** and thus small lesion detectability. • **Improved timing resolution**, see Time-of-flight (TOF) below.
**Time-of-flight (TOF)** • Including the position of the annihilation on the line of response (LOR) allows for better discrimination between random and true coincidence events. Random events with TOF detection will often result in placement of the event outside the imaged body, which will not contribute to the noise inside the body and reduce the noise in the image. Thus **increasing the signal-to-noise ratio**. • The higher peak noise equivalent count rate results in a **better and more uniform convergence** of the reconstruction algorithm. This improves the quantitative accuracy and the lesion detectability, especially in obese patients.
**Point-spread function modeling** • Point-spread function modeling includes the physical processes that cause image degradation, including positron range, photon non-collinearity, and detector-related effects (including crystal widths, intercrystal scattering, and intercrystal penetration). This results in **noise reduction** and **spatial resolution uniformity**.
**Bayesian penalized likelihood reconstruction** • The reconstruction introduces a term which penalizes noisy solutions that increase the variation between neighboring voxels. Therefore, the algorithm can run until full convergence, which leads to a better quantitative accuracy. • By penalizing noisy solutions the signal-to-noise ratio is decreased, which improves image contrast in particular for small lesions.

*The bold words summarize the key benefits of a particular technical breakthrough*.

Traditionally hampered by a rather modest image resolution, PET imaging has seen significant technological advances over the last decade resulting from improvements in detector hardware and advances in image reconstruction algorithms. Most notably, the introduction of (digital) solid-state photodetectors, time-of-flight (TOF) image reconstruction, point-spread-function (PSF) modeling, and Bayesian penalized likelihood (BPL) based reconstructions, have contributed to higher image quality.

Progress in detector design includes the introduction of (digital) solid state photodetectors like the digital silicon photomultiplier (SiPM) (11). These have contributed to a higher image quality, improved small lesion detection, and allow for a lower administered activity of FDG, reducing patient radiation exposure. The improvements in reconstruction algorithms can be briefly summarized as follows. PET relies on the detection of two coincident photons generated by positron annihilation events to determine the location of the source. This requires multiple detected photon pairs within the circular PET detector. However, time-of-flight reconstructions improve this process by also taking into account the time difference between the detection of both annihilation photons, requiring less photon pairs for equivalent information on the source position. The use of TOF image reconstruction improves the signal-to-noise ratio (in particular in obese patients), improves the detection of small lesions, and enables imaging with lower injected activities (12). In addition, point-spread-function modeling addresses the physical characteristics of the different components of the PET detector system improving the uniformity of the spatial resolution and reducing image noise (13).

Image quality can be further improved with the use of latest generation image reconstruction algorithms. For example, the Bayesian penalized likelihood method results in improved image quality in particular for small lesions. The image resolution of PET systems is usually expressed using the standardized “full width half maximum” (FWHM) methodology, meaning that two ideal point-sources will appear separate in the image when they are a distance greater than the FWHM apart. For the latest generations whole-body systems this ~3.5–4 mm in the transaxial axis (14, 15), with a theoretical physical lower limit of clinical PET imaging systems of ~2 mm (16).

## Clinical Impact of Newer PET/CT Designs

Taken together, the type of PET/CT scanner and the chosen method of image reconstruction nowadays more than ever determines the quality and potential artifacts of the generated images. In clinical practice this is especially important when patients are scanned using different scanners during follow-up, as the observed changes in tumor metabolic activity may be real or caused by differences in the used devices or reconstruction settings.

In the coming years, it is expected that further technological advances will change clinical practice and revolutionize the PET/CT arena. In particular, the first full-body PET/CT devices have now become commercially available, enabling unprecedented image quality with very small amounts of activity (25 MBq [0.7 mCi] or less) and scan times of ~1 min (17). This represents reductions of 80–90% in both injected activity and scan duration compared to previously available scanners.

## From Qualitative to Quantitative Interpretation

In routine clinical practice, the mainstay of PET/CT examinations are reported by visual qualitative assessment of regional tracer distribution where both the intensity and pattern of uptake will guide the judgment on calling a lesion benign or malignant. Obviously, this type of assessment is prone to error due to both technical and reader related issues, and may be associated with considerable inter-rater variability depending on expertise. Yet, due to the physical characteristics of the PET technique, the image data is inherently quantitative and early-on the SUV emerged as semi-quantitative measure of tracer uptake, becoming the predominant metric for quantification of FDG-PET/CT scans. Indeed, oncology PET literature data is entrenched with proposed SUV thresholds to distinguish benign from malignant disease.

However, the SUV is not without flaws as this metric is vulnerable to many sources of unwanted variability, including patient preparation and characteristics, scanner capabilities, and calibration, image reconstruction settings, and tumor volume-of-interest (VOI) delineation techniques (18). Biologic factors that result in artificially lower SUVs include lower body fat percentage, higher blood glucose level, and shorter post-injection uptake time (19). Therefore, an SUV should always be interpreted with caution if information on these factors is lacking. Recognizing these issues, it was recommended early-on that imaging should be performed on the same scanner using the same image acquisition and reconstruction protocols when serial SUV measurements are used to assess treatment response, as well as meticulous attention to accurate determination of the administered radiopharmaceutical activity (19). While this may be feasible in a single-center setup, this becomes much harder when collaborating in a group of hospitals or in the context of a multi-center clinical trial. To overcome these limitations, a number of quality assurance and control measures have been proposed together with a framework for the harmonization of FDG-PET/CT acquisition and reconstruction.

In a recent multi-center study of FDG-PET/CT surveillance 12 weeks after concurrent chemotherapy, it was demonstrated that using an SUV threshold (SUV_70_ 2.2) performed equally well as visual analysis to detect nodal relapse, but required that SUV was measured using standardized acquisitions and reconstructions. Comparing with a historical control cohort of patients imaged in non-standardized conditions, the same authors showed that SUV ratios consisting of lesion uptake and a background region (e.g., the liver) may help to reduce some of the variability introduced by using non-standardized protocols (20). This may be explained by the fact that systematic system errors causing over- or underestimation of SUVs are canceled out to some extent by using relative ratios.

## Quality Management in Nuclear Medicine

Ideally, the quality measures described below are implemented within the context of a quality management system that standardizes the process to guarantee consistency in providing high level services to patients, referring physicians, and other stakeholders in a safe environment. To this end, the International Atomic Energy Agency (IAEA) has developed the Quality Management Audits in Nuclear Medicine Practices (QUANUM) framework to guide nuclear medicine services to achieve this goal (21).

## Quality Assurance and Control of FDG-PET/CT

**Figure 1:**
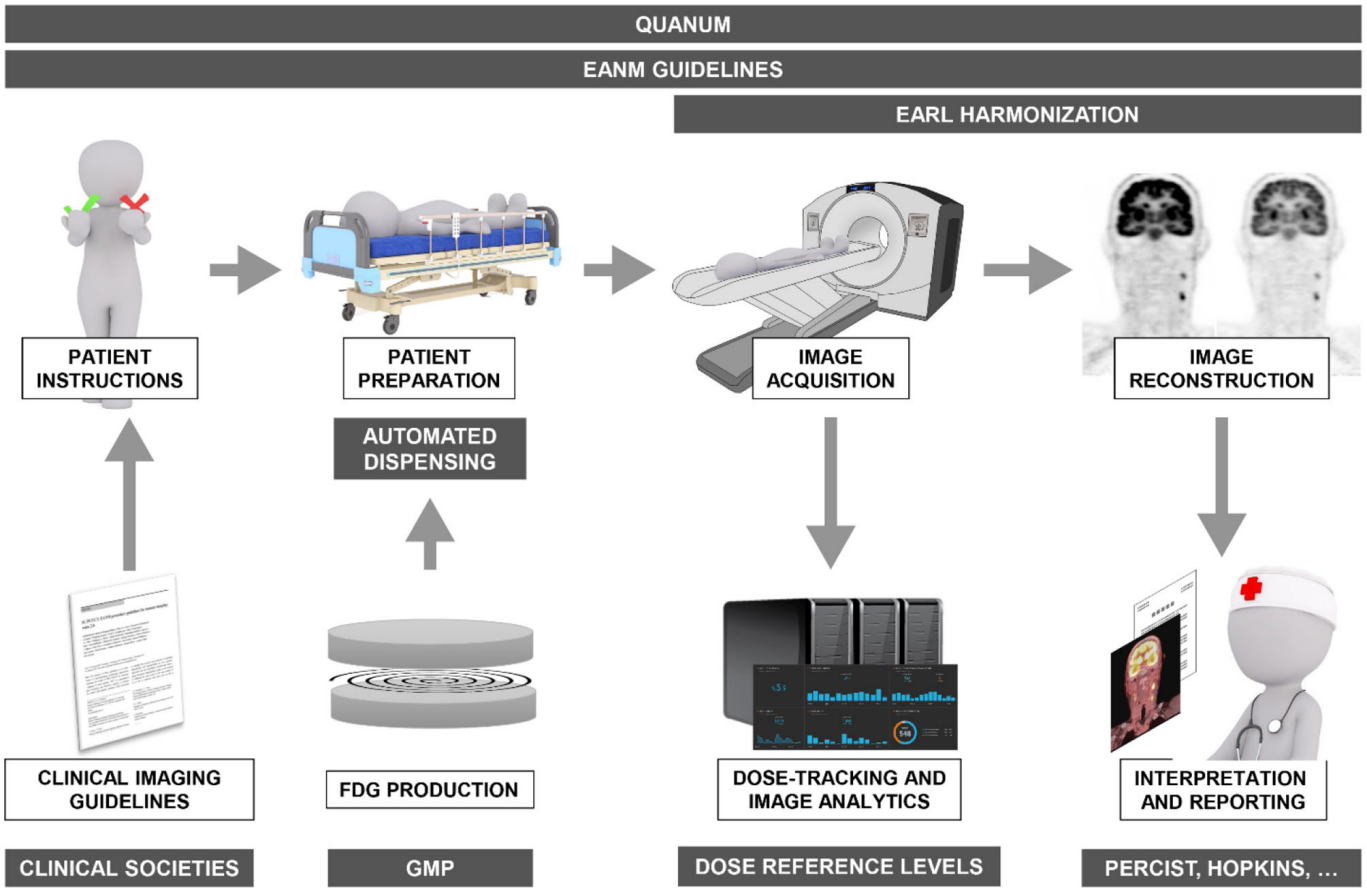
Overview of the various quality assurance/control mechanisms (gray boxes) and frameworks in operation today covering the entire spectrum from FDG synthesis, patient instructions and preparation, to image acquisition, reconstruction, and reporting to ensure optimal diagnostic accuracy of FDG-PET/CT imaging in oncology.

Quality assurance (QA) is the collective set of pro-active measures taken to ensure the quality of the entire process involved in performing the diagnostic study. It aims to prevent any errors or issues with the examination that may affect its quality by focusing on this process. In contrast, quality control (QC) describes the set of *post-hoc* activities that are carried out after the examination has been performed to ensure its quality, with the aim of identifying and correcting any errors or issues. As discussed previously, the quantification of PET data is particularly susceptible to variations in administered activity, tracer incubation times, scanner characteristics and image reconstructions settings. Therefore, it is not surprising that many of the measures listed below will aim to reduce variability in procedures by standardizing these parameters (Figure 1).

### Tracer Production

The routine synthesis of FDG is semi-automated and multiple commercial systems are available to produce this radiopharmaceutical for just-in-time delivery in a way that is fully compliant with Good Manufacturing Practice (GMP) (22, 23). Using this approach, FDG can nowadays be reliably synthesized meeting the quality requirements as outlined in various pharmacopeia (24). As a consequence, issues in the production of FDG as cause for errors in PET/CT scans have become virtually non-existent.

### Patient Preparation

Real-world data confirms that there is considerable heterogeneity in clinical routine practice of FDG-PET/CT imaging with respect to the imaging protocol used (25). This is probably inspired by the numerous studies reporting alternate patient preparation or scanning protocols over the years (26–30). In order to reduce this variability and possible errors introduced by this practice, the European Association of Nuclear Medicine (EANM) has published a detailed guideline for FDG-PET/CT imaging in oncology, including recommended acquisition protocols (31). While a detailed discussion is beyond the scope of this paper, the EANM guideline provides useful recommendations on:

- Food and drink consumption before the study
- Physical activity prior to the study
- Management of patients with diabetes
- Management of serum glucose level before tracer administration
- Measures to reduce physiologic tracer uptake in brown adipose tissue
- Hydration status
- Administered activity
- Suggested environmental conditions during the FDG uptake phase
- Patient positioning during the scan.

### Automated Dispensing and Injection

FDG is usually delivered as a multi-dose vial and subsequently dispensed and administered to the patient. This means that a manual procedure is required to remove the desired amount of activity from the vial and inject this into the patient. Not only does this repeated manual dispensing and administration expose the imaging technician to a significant amount of radiation, it also introduces the possibility of unintended over- or underdosing by errors in using the dose calibrator or unintentional residue left in the syringe or tubing.

To overcome this source of error, automated dispensing and injection systems have been developed. These systems have a built-in reservoir for storing FDG in a sterile and shielded way, contain a dose calibrator connected with the reservoir, and have a system of tubing and pumps that are able to deliver a requested amount of activity to a shielded syringe or device ready for injection into the patient. Data has shown that these systems are accurate, deliver activities for injection within a 3% margin of that requested, combined with reductions in the radiation exposure to the hands and fingers of technologists of 80–94% compared to manual dispensing and injection (32, 33).

### Acquisition Protocol

The EANM guideline also gives guidance for the acquisition protocol (31), both for the PET and CT parts. Focusing on head-and-neck cancer, it is noteworthy to highlight the recommended two-step protocol to reduce artifacts in the head-and-neck region caused by the patient's arms when imaging in the arms up position: first the head-and-neck portion is imaged with the arms down, followed by a scan from apex of the lung through the mid-thigh with the arms up (34). In addition, acquisition of an additional dedicated head-and-neck image series with a higher PET resolution than that of the whole-body image set together with a contrast enhanced CT is recommended in the staging of head and neck cancer as it improves the detection of small lymph node metastases (35).

When the PET/CT study will be used for radiation planning, the patient positioning should mimic that of the radiotherapy set-up as closely as possible, including the use of a radiotherapy table top, laser alignment, immobilization devices, and measures (36). Especially for the head-and-neck region, immobilization techniques should be used to prevent movement of the head between the acquisition of the CT scan and the PET images. Indeed, while PET/CT scanners are hybrid imaging devices, the CT and PET study are not acquired at the same time, but rather in a sequential fashion. Any patient movement between the two acquisitions will result in misregistration artifacts when viewing the fused images and may lead to errors in lesion localization.

### Device Calibration

System calibrations typically include a daily check, periodic detector normalization, two to three dimensional radioactivity concentration calibration, as well as other parameters considered critical for quality assurance. A recent interim report from the IAEA QUANUM audits presenting results collected mostly in South America and Asia noted that the checklists covering quality control for imaging equipment showed the lowest values of conformance (68.3%), highlighting the need for continued attention in this area (37). Data from Austria obtained outside the scope of QUANUM confirm that the use of standardized QC procedures is a point for improvement in order to increase quantitative accuracy across PET/CT centers (25).

### Harmonization

The most important contribution to the standardization of quantification of PET/CT across centers has without doubt been the harmonization effort set-up by the European Association of Nuclear Medicine (EANM) through their EANM Research Ltd (EARL) subsidiary. This accreditation program started in 2010 and has since been endorsed by the European Organisation for Research and Treatment of Cancer (EORTC) Imaging Group. Other efforts with similar goals have been initiated by American Society of Nuclear Medicine (38) and international consensus protocols have also been published (39).

**Figure 2:**
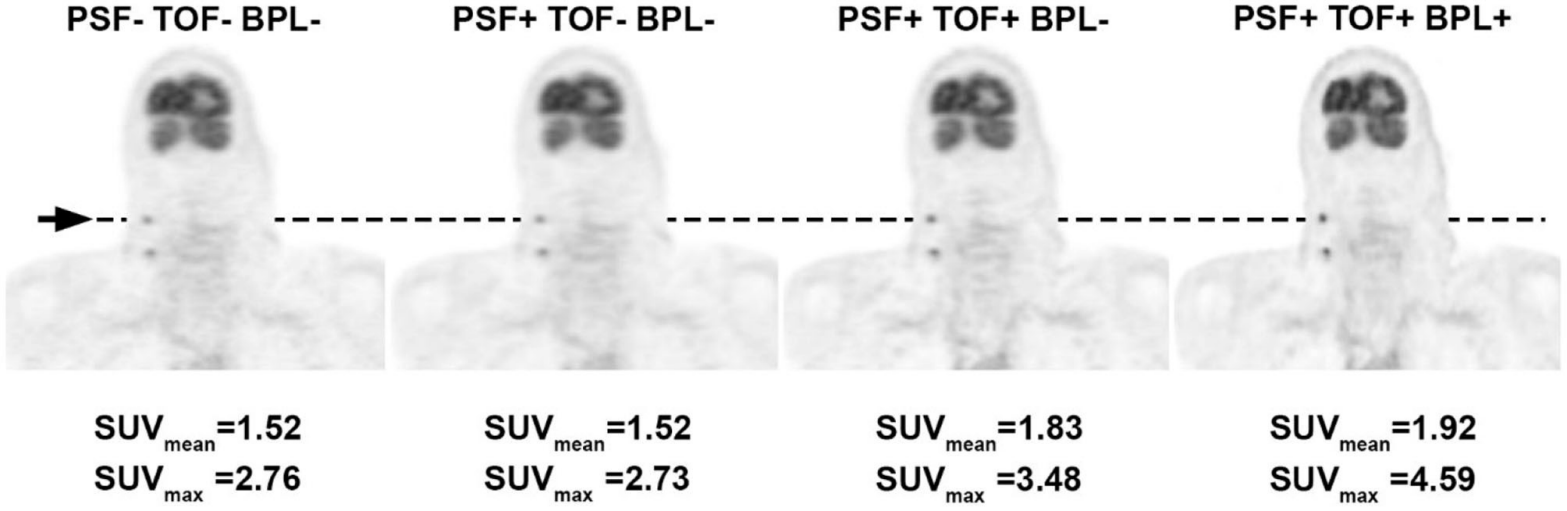
Illustration of the upward creep in SUV values resulting from the use of recently introduced novel reconstruction techniques. The same FDG PET dataset in a patient with cervical lymph node metastases of a head-and-neck malignancy was reconstructed using a traditional iterative algorithm and subsequently with additional image improving techniques: PSF, point-spread-function modeling; TOF, time-of-flight; BPL, Bayesian penalized likelihood. SUV values are presented for the same cervical lymph node and measured in the same 1 cm^3^ volume-of-interest (arrow), showing a clear increase in value, in particular for the maximum SUV of the lesion.

The specific aim of EARL is to ensure the exchangeability of quantitative PET/CT metrics (like SUV) in a multicenter setting or to improve the implementation of quantitative interpretation criteria (such as SUV thresholds) in routine clinical practice (40). While a detailed description of the EARL protocol is beyond the scope of this text, it has been shown that compliance with EARL is feasible and able to resolve most causes of errors in quantitative PET measurements when combined with adherence to the FDG-PET/CT imaging guidelines (41). Designed in 2010, the EARL currently do not cover newer systems, that have been shown to produce higher maximum SUV values (Figure 2) resulting in discordant treatment response assessments (42). Based on these findings, an update of the EARL system has recently been proposed to include modern PET/CT equipment to mitigate these discrepancies (43).

### Standardized Reporting

With the acquisition process and image reconstruction being harmonized, the next source of variability in FDG-PET/CT imaging is the interpretation of the images by the reading physician. In particular in the setting of treatment response assessment, the need for standardization of reporting was recognized early on. In 1999 the EORTC criteria were published, based essentially on changes in SUV (44). This was later superseded PERCIST, which also uses quantification as means to standardize the interpretation of response (45). New concepts introduced by PERCIST were:

- Checking variability of uptake between scans in a fixed background region (i.e., the liver) to assess whether comparisons between scans are appropriate
- Establishing a threshold of minimum uptake in a target lesion on the baseline scan required for a meaningful comparison
- The use of lean body mass adjusted standardized uptake value (SUL) to minimize the impact of body weight
- Selection of SULpeak (i.e., the highest average SUL in a sphere with predefined size contained in the lesion) rather than SUVmax (i.e., the hottest pixel in the lesion) as outcome metric.

In patients with head-and-neck squamous cell carcinoma receiving FDG-PET/CT before and ~3 months after concurrent chemoradiotherapy, response as assessed with PERCIST was found to be a predictor of progression-free and overall survival (46). This has prompted interest in using FDG-PET/CT earlier during treatment to identify patients who may not respond. For example, a recent study suggested that FDG-PET/CT performed 14 days after the start of neo-adjuvant chemotherapy in patients with locally advanced disease was able to identify patients with poor outcomes, based on an increase in regional lymph node maximum SUV and insufficient decrease in primary tumor uptake after 2 weeks of treatment (47). Currently, the PERCIST thresholds (decrease ≥30%) do not vary according to the number of treatment cycles received (i.e., mid-treatment or end-of-treatment), which may change in subsequent versions (48).

Specifically for head-and-neck cancer response assessment, Marcus et al. proposed the Hopkins 5-point interpretation criteria to assess locoregional response after chemoradiotherapy. This system compares the tracer uptake of residual lesions with that of the activity in the internal jugular vein or the liver. Only uptake higher than that of the liver is deemed to be residual malignant disease (49, 50). The clinical value of the Hopkins scoring system was validated in a prospective multicenter study, showing that the system is reliable when used for FDG-PET/CT surveillance 12 weeks after concurrent chemoradiotherapy (51). Of note, this study did highlight that the sensitivity of the Hopkins scoring system was strongly time dependent, meaning that while it detects residual disease in patients who relapse up to a 9-month horizon after imaging with high sensitivity, it is less able to do so for patients who relapse later on, possibly because residual disease is either still below the detection threshold or metabolically inactive at the 12-week imaging timepoint. Therefore, clinicians may consider a second surveillance scan at ~12 months after the end of chemoradiotherapy.

### Dose-Tracking and Imaging Analytics Platforms

Over the last years, a number of platforms have been introduced allowing automatic analysis of imaging studies on a hospital-wide scale using data stored in the Picture Archiving and Communication System (PACS), presenting useful metrics in a convenient dashboard-style interface. For example, using this technology patient radiation exposure from the CT part can be monitored on a population level for compliance with national dose reference levels (DRL) (52). For the PET part, conformance with the EANM imaging guideline can be checked and systematic sources of error can more easily be identified and subsequently corrected to prevent future errors. With advances in artificial intelligence it can be expected that the ability of these platforms to detect deviations from imaging protocols will increase, and where they are now primarily used to detect issues after the facts, it is not inconceivable that they may evolve to gatekeepers running in the background that are able to prevent errors before they occur.

## Conclusions

FDG-PET/CT has evolved to a clinically important imaging modality in head-and-neck cancer with a significant impact on patient management and outcome. Subsequent technical advances have increased the capabilities, but also the complexity, of the latest PET/CT scanners. Combined with a desire to move to more quantitative image analysis, it has become apparent that rigorous quality assurance is required spanning the entire workflow from tracer synthesis to patient preparation, image acquisition and reconstruction, and interpretation. Thanks to a coordinated effort over the last decade of industry, academia, and professional societies the frameworks that allow harmonization of FDG-PET/CT are in existence today and should be implemented across the board in order to consolidate PET/CT as leading standardized functional imaging technique.

Referring to the subtitle of this review: recent technical advances may usher in the next homerun for PET/CT, but only if we control for the potential pitfalls by rigorous harmonization and conforming practice to applicable guidelines. If not, we risk diluting the tremendous potential of the latest generation of PET/CT scanners and loose the opportunity to put a prestigious run on the scoreboard for this great technique.

## Author Contributions

TV performed the literature search and wrote the manuscript. SD contributed to the physics parts of the text. LC reviewed the manuscript. All authors contributed to the article and approved the submitted version.

## Conflict of Interest

The authors declare that the research was conducted in the absence of any commercial or financial relationships that could be construed as a potential conflict of interest.
